# Resilience Predicts the Trajectories of College Students’ Daily Emotions During COVID-19: A Latent Growth Mixture Model

**DOI:** 10.3389/fpsyg.2021.648368

**Published:** 2021-03-30

**Authors:** Li Zhang, Lei Wang, Yuan Liu, Junyi Zhang, Xiaoying Zhang, Jingxin Zhao

**Affiliations:** ^1^Faculty of Education, Shandong Normal University, Jinan, China; ^2^School of Psychology, Shandong Normal University, Jinan, China

**Keywords:** latent growth mixture, trajectories, positive affect, negative affect, resilience

## Abstract

The objective of this study was to examine the association between resilience and trajectories of college students’ negative and positive affect during the COVID-19 pandemic. A total of 391 college students recruited from China completed a daily online negative and positive affect scale for 1 week, and their resilience was also measured. Profiles of brief trajectories of negative and positive affect over time were identified using the latent growth mixture model, and the effect of resilience on these trajectories was further explored. Two latent profiles of negative affect were found: a constant high negative affect profile and a slowly decreasing low negative affect profile, while three latent profiles of positive affect were identified: a slowly increasing high positive affect profile, a rapidly decreasing medium positive affect profile, and a constant medium positive affect profile. The optimism dimension of resilience predicted the membership in the various profiles significantly, whereas the prediction of tenacity and strength dimensions of resilience was not significant. Activities that promote resilience, especially optimism, should be included to improve the daily emotions of college students during COVID-19.

## Introduction

The COVID-19 outbreak in December 2019 has rapidly spread around the world and developed into a global public crisis that has caused a significant threat to humans. The increasing menace of the COVID-19 pandemic has not only hindered people’s travel plans, social interaction, and economic development, but has also presented psychological challenges. Recent studies show that the COVID-19 pandemic is correlated with mental disorders (e.g., anxiety, depression, perceived stress, suicides) among the general population around the world (Dai et al., 2020; Galea et al., 2020; Goyal et al., 2020; Sherman et al., 2020; Shi et al., 2020; Wang C. et al., 2020). As the most dynamic group, college students are also suffering from emotional distress (e.g., worry, fear, sadness) during COVID-19 (Kecojevic et al., 2020; Odriozola-González et al., 2020). Their emotional experiences during COVID-19 tend to be heterogeneous (Riehm et al., 2020), with some college students displaying resilience and others displaying vulnerability. However, the associations between resilience and the heterogeneous trajectories of college students’ daily emotions are poorly understood. This study was designed to go beyond previous research by examining the heterogeneous trajectory of college students’ daily negative and positive affect, and investigating the prediction of resilience on college students’ trajectories of daily emotion.

Since the COVID-19 outbreak, lockdown, local traffic, and other measures have been adopted to prevent the spread of the epidemic in China. These measures have effectively controlled the epidemic and reduced people’s negative affect to a certain extent, but they also disrupt day to day routine activities that can bring new negative experiences (Brooks et al., 2020). For college students, they not only face these enormous disruptions to daily life, but also encounter additional challenges, such as online learning, online exams, etc. Some college students may have difficulties accessing a computer and the internet at home (Kecojevic et al., 2020). These dramatic changes and challenges potentially influence college students’ emotional experiences. Thus, college students’ emotional experiences have become an interest in research during COVID-19. For example, Kecojevic et al. (2020) found that college students showed higher levels of depression and anxiety during COVID-19. Charles et al. (2021) reported that college students’ mood disorder symptoms increased during the COVID-19 lockdown. Fu et al. (2021) found that 41% of 89,588 college students reported anxiety symptoms. However, the limited research examining the impact of COVID-19 on the emotional experiences of college students has primarily focused on pathological outcomes; there has been a dearth of work on daily emotion related to COVID-19 among college students. One primary aim of the present study was to investigate the variance of daily negative affect of college students during COVID-19.

Increasing negative affect does not mean the non-existence of positive affect during COVID-19. Positive affect and negative affect are distinct dimensions of human emotional experiences (Diener et al., 1985; Fabes et al., 2002), which can be measured separately, and varied independently (Russell and Carroll, 1999; Larsen et al., 2001). Indeed, empirical evidence suggests that college students do experience positive affect during an epidemic. For example, Monorom and Mongkut (2020) found that college students who studied or did exercise for longer at home reported more happiness during the COVID-19 pandemic outbreak. Maher et al. (2020) found that college students also experience positive affect (e.g., proud, excited, interested) during COVID-19. However, research into college students’ positive affect is still rare, let alone their daily positive affect during the COVID-19 pandemic. Thus, the current study fills this gap by examining college students’ positive affect in daily life during COVID-19. Examination of college students’ daily positive affect during COVID-19 might not only help understand college students’ daily emotional experiences during periods of public health events, but also act as intervention targets for supportive care services offered to college students at risk of poor emotion mood.

Apart from focusing on both the daily negative and positive affect of college students, it is also important to test the heterogeneous trajectories of daily emotional experiences over time during COVID-19. Individual differences among daily emotion changes are more complex than simply differences in mean levels of emotions (Nandi et al., 2009). There may be subpopulations of college students who may differ in trajectories of daily emotional experiences (Brondino et al., 2020). For instance, some college students may experience high levels of positive affect over time, whereas some college students may show high levels of negative affect. Additionally, some college students may change emotions quickly, while others are insensitive to it (Eaton and Funder, 2001). Characterizing heterogeneous trajectories of college students’ daily emotional experiences may inform an understanding of the development of personal emotion distress during COVID-19. Latent growth mixture modeling (LGMM) is a person-centred approach that can model heterogeneity growth trajectories by classifying individuals into latent groups (Muthén and Shedden, 1999). There is some evidence showing that trajectories of college students’ daily emotional experiences are heterogeneous. For example, Brondino et al. (2020) investigated trajectories of positive affect for a week among 108 college students using LGMM, and three heterogeneous classes were identified: a constantly high positive affect profile, an increasing positive profile, and a decreasing positive profile. However, the trajectories of negative and positive affect during the pandemic might be different from that of routine life. Using LGMM, the present study assessed the distinct latent trajectories of daily negative and positive affect in college students during COVID-19.

An additional important question concerns which predictors differentiate the trajectories of daily negative and positive affect in a heterogeneous group of college students during COVID-19. Resilience refers to a set of personal attributes facilitating individuals’ positive adaption within negative contexts and traumatic events, which may include the attributes of tenacity, optimism, and strength (Connor and Davidson, 2003; Fergus and Zimmerman, 2005; Yu and Zhang, 2007). Several studies have directly tested the hypothesis that resilience was associated with college students’ emotional experiences. For example, Zhao et al. (2020) found that there was an association between personal resilience and positive affect. Kaye-Kauderer et al. (2019) found a correlation between resilience and negative emotion (e.g., anger, sadness, anxiety). These findings suggest that resilience is a personal resource which may produce adaptation and protect individuals from the severe emotional consequences of the pandemic. However, very little is known about associations between resilience and different trajectories of daily negative and positive affect during COVID-19 in college students. To address this gap, the present study examined the role of resilience in differentiating latent trajectories of negative and positive affect in college students during COVID-19.

In addition, it is necessary to examine potential gender difference in trajectories of daily negative and positive affect and in the association between resilience and these trajectories. Researchers have previously found gender differences in daily emotional experiences (Larson et al., 2002; Silk et al., 2003). For instance, Silk et al. (2003) found that adolescent girls experienced more negative affect; conversely, Larson et al. (2002) found that adolescent girls experienced more positive emotions. However, others found no gender difference (Weinstein et al., 2007). Considering these mixed findings and the fact that little work focuses on gender difference in trajectories of daily negative and positive affect, further studies are needed to investigate how trajectories of daily negative and positive affect over time relate to gender. Furthermore, several studies have found that females experienced more adverse effects and lower resilience than males (Bonanno et al., 2007). However, whether gender differences exist in the relation between resilience and daily negative and positive affect is unclear, as evidence uncovered by extant studies is limited. The present study extends previous research by examining gender differences in association between resilience and trajectories of emotional experiences of college students.

Using a person-centred approach, the major aim of this study was to identify heterogeneous trajectories of daily negative and positive affect over time, and to examine the relation between college students’ resilience and heterogeneous trajectories during COVID-19. The gender differences in trajectories of daily negative and positive affect, and in the relation between resilience and trajectories of daily negative and positive affect were also examined. Specifically, we hypothesized that: (a) different trajectories of daily negative and positive affect in college students during COVID-19 would be found; (b) resilience would be positively correlated with positive affect trajectories, while negatively correlated with negative affect trajectories; and (c) the trajectories of negative and positive affect, and relation between resilience and such heterogonous trajectories would be different for males and females.

## Materials and Methods

### Participants

The participants in this study were recruited from fifteen provinces in China. A total of 407 college students were invited as the initial sample. Among these, 399 college students also took part in the follow-up assessments during the following consecutive 7 days. Of participants who provided data on all 7 days, eight college students who returned incomplete questionnaires were excluded. Thus, 391 college students (*M* = 20.77 years, *SD* = 1.01 years) were eventually selected for the study, including 248 females and 151 males. The response rate was 96.07% (391/407). None of the participants had an observable physical or developmental disability. The majority of the parents of participants had an educational level of high school to college or higher and a job with a stable income. An independent *t*-test was used to compare college students who participated in all 21 data collection waves to those who dropped out of the study between these waves on all study variables at the first wave. The results revealed that those who participated in the study for 21 waves were not significantly different from those who dropped out of the study after the first wave.

### Procedures

The procedures of this study were approved by the Institutional Review Board of Shandong Normal University. Informed consent was obtained from all the participants prior to each data collection. Daily data was collected using the experience sampling method (ESM, Csikszentmihalyi et al., 1977), which was used to assess college students’ daily emotional experiences in their living environment. The data collection in this study took place three times per day (10:00 a.m. ∼ 2:00 p.m., 2:00 p.m. ∼ 6:00 p.m., 06:00 p.m. ∼ 10:00 p.m.) for seven consecutive days (from Monday to Sunday) during the period between March 23 and 29, 2020. During this period, COVID-19 was basically controlled and in a stable stage in China. Each data collection took approximately 3 min. In total, participants filled out 21 momentary assessments.

Before data collection started, psychology students provided training to the participants on the survey completion with smart phones acting as data-collecting devices. The training included the study propose, process, and how to use the survey internet connection. Importantly, they also informed the participants to complete the survey based on their emotional state at the time they received the QQ message. Every day’s time interval between QQ messages was at least 30 min. The survey became inactive if participants did not respond to a signal within 10 min. All the data collection followed the same procedure.

### Measures

#### Demographic Variables

The demographic information about the individual and their family were measured. Individual information included gender (i.e., female and male), grade (i.e., freshman, sophomore, junior, and senior grade), age, and region of residence. Family information included the degree of parental education (i.e., primary school or below, junior high school, senior high school, technical secondary school, undergraduate, master, doctor), parental occupation (i.e., full-time job with income, part-time job with income, unemployment but looking for a job, or other conditions such as taking care of family or retirement), and monthly income of family. The total scores of the three family-level demographic variables were calculated as the social economic status (SES) of the family (Chung, 2015).

#### Daily Negative and Positive Affect

ESM data were collected with smart phones for seven consecutive days, three times a day. The ESM questionnaires examined participants’ daily negative and positive affect through a 11-word list selected on the basis of two resources: (a) four of the six basic emotions, including joyful, angry, afraid, and sad (Watson et al., 1988; Levenson, 2011), and (b) emotions closely correlated with the current COVID-19 pandemic and other similar epidemics, including anxious, lonely, peaceful, and so on (Tausczik et al., 2012; Chou and Budenz, 2020; Moroń and Biolik-Moroń, 2020). Among them, seven words were about negative affect (i.e., sad, angry, irritable, afraid, lonely, anxious, boring), and the other four words were about positive affect (i.e., proud, contented, peaceful, joyful). Participants reported how strongly they felt through a five-point Likert scale (1 “not at all” to 5 “extremely strong”). The seven negative words added up to the scores of negative affect, and the four positive words contributed to the scores of positive affect, with higher scores indicating a stronger degree of negative or positive affect. The Cronbach’s α coefficients of negative affect ranged from 0.81 to 0.91. The Cronbach’s α coefficients of positive affect ranged from 0.63 to 0.80.

#### Resilience

The Connor-Davidson Resilience Scale (CD-RISC, Connor and Davidson, 2003; Yu and Zhang, 2007) was used to assess participants’ three different dimensions of resilience: tenacity (13 items, e.g., “when things look hopeless, I don’t give up”), strength (eight items, e.g., “coping with stress strengthens”), and optimism (four items, e.g., “see the humorous side of things”). Participants answered the items through a five-point Likert scale (0 “not true at all” to 4 “true nearly all of the time”). The total scores were calculated, with higher scores indicating higher levels of resilience. The Cronbach’s α coefficients of the tenacity, strength, and optimism dimensions were 0.88, 0.81, and 0.57, respectively.

### Data Analysis

#### Preliminary Analyses

In order to describe the changes of college students’ daily emotional experiences over 7 days, the total scores of negative and positive affect measurements three times a day were separately calculated, which was an indicator of college students’ daily negative and positive affect. Descriptive analysis was performed to describe the means and standard deviations of each study variable. Paired samples *t*-tests were used to explore the individual differences in daily positive and negative affect over 7 days. Independent samples *t*-tests were used to investigate the gender differences in resilience and daily emotion. Correlation analysis was used to describe the relation between resilience and daily negative and positive affect.

#### Latent Growth Mixture Models

To explore the trajectories of negative and positive affect, LGMMs were conducted separately for the daily positive and negative affect with gender and SES as covariates. The latent growth mixture model was an approach used to identify the trajectories of different subgroups, with intercept representing the initial level, and slope representing the change rate. For both daily negative and positive affect, the time scores of the intercepts were all fixed as one, indicating that the intercept was time-invariant. The time scores of the slopes were fixed as 0–6 for the seven waves of data, indicating that the slope varied linearly as time. A series of LGMMs were conducted to identify the profiles of negative affect trajectories and positive affect trajectories. The model fitting the data best was selected according to the following fix indexes: Bayesian information criterion (BIC), Akaike information criterion (AIC), sample size-adjusted Bayesian information criterion (aBIC), entropy, Lo–Mendell–Rubin adjusted likelihood ratio test (LMR-LRT), and bootstrap likelihood ratio test (BLRT). A lower BIC, AIC, and aBIC indicated a better fit. Higher entropy indicated greater precision of classification. Significant LMR-LRT and BLRT indicated better fit of the model than a model with one less class. Full information maximum likelihood estimation (FIML) was used to address the missing data. Analyses were conducted through Mplus 8.3.

#### Logistic Regressions

Logistic regressions were used to explore the relation between the likelihood of membership into the various trajectories class, tenacity, strength, optimism, and gender. It should be noted that the dependent categorical variable consisted of unordered categories. In this case, the log-odds of membership in each category were calculated relative to a chosen reference category (Wiesner and Windle, 2004). Significant predictors (*p* < 0.05) were entered into the logistic regression. Insignificant predictors (*p* > 0.05) were excluded.

## Results

### Descriptive and Bivariate Analysis

Table 1 shows the means and standard deviations of the key variables. Paired samples *t*-tests showed that there was a significant difference in individual’s daily negative and positive affect. Specifically, individuals had more negative affect than positive affect from Monday to Saturday (*p*s <0.10). Independent samples *t*-tests showed that, males reported higher levels of tenacity, strength, optimism, and positive affect from Tuesday to Friday compared with girls (*p*s <0.05). However, there were no significant gender differences in negative and positive affect at other times (*p*s >0.05).

**TABLE 1:** Means and standard deviations for main study variables (*N* = 391).

**Variables**	**T1**		**T2**		**T3**		**T4**		**T5**		**T6**		**T7**	
	***M***	***SD***	***M***	***SD***	***M***	***SD***	***M***	***SD***	***M***	***SD***	***M***	***SD***	***M***	***SD***
Positive affect	2.82	0.70	2.62	0.70	2.58	0.74	2.51	0.73	2.57	0.75	2.54	0.81	2.60	0.77
Negative affect	1.87	0.60	1.68	0.60	1.57	0.58	1.57	0.59	1.54	0.61	1.53	0.61	1.50	0.60
Tenacity	2.49	0.57												
Strength	2.76	0.55												
Optimism	2.58	0.58												

**T1 = Monday, T2 = Tuesday, T3 = Wednesday, T4 = Thursday, T5 = Friday, T6 = Saturday, T7 = Sunday.**

The bivariate correlations for the main study variables are presented in Table 2. As shown, tenacity, strength, and optimism were significantly and positively correlated with positive affect on all days (*p*s <0.001). Strength was negatively correlated with negative affect on all days (*p*s <0.05). Except for Friday, optimism was negatively correlated with negative affect on the other days (*p*s <0.05). Tenacity was only negatively correlated with negative affect on Monday, Tuesday, and Saturday (*p*s <0.01). There was a significantly positive correlation between tenacity, strength, and optimism (*p*s <0.01). Furthermore, the smallest correlation existed between negative and positive affect among the 7 days (*p*s >0.05).

**TABLE 2:** Bivariate analysis results of all the study variables (*N* = 391).

**Variables**	**1**	**2**	**3**	**4**	**5**	**6**	**7**	**8**	**9**	**10**	**11**	**12**	**13**	**14**	**15**	**16**	**17**
1. T1 positive affect	–																
2. T2 positive affect	0.72***	–															
3. T3 positive affect	0.63***	0.76***	–														
4. T4 positive affect	0.66***	0.76***	0.78***	–													
5. T5 positive affect	0.62***	0.69***	0.74***	0.81***	–												
6. T6 positive affect	0.52***	0.70***	0.72***	0.79***	0.82***	–											
7. T7 positive affect	0.53***	0.63***	0.68***	0.75***	0.79***	0.81***	–										
8. T1 negative affect	−0.23***	−0.14*	−0.11*	−0.14*	−0.14**	−0.14*	–0.09	–									
9. T2 negative affect	−0.12*	−0.15**	–0.01	–0.02	–0.05	–0.09	0.01	0.69***	–								
10. T3 negative affect	–0.07	–0.01	–0.07	0.04	–0.01	–0.03	0.03	0.60***	0.74***	–							
11. T4 negative affect	–0.03	0.05	0.03	–0.03	0.01	–0.02	0.06	0.59***	0.70***	0.79***	–						
12. T5 negative affect	–0.02	0.06	0.06	0.06	–0.08	–0.02	0.03	0.52***	0.65***	0.75***	0.78***	–					
13. T6 negative affect	–0.05	–0.01	0.03	0.03	–0.06	−0.11*	0.02	0.52***	0.66***	0.73***	0.77***	0.83***	–				
14. T7 negative affect	0.04	0.08	0.07	0.08	0,01	–0.02	–0.02	0.50***	0.64***	0.77***	0.80***	0.86***	0.83***	–			
15. Tenacity	0.37***	0.28***	0.26***	0.27***	0.26***	0.20***	0.22***	−0.19***	−0.19***	–0.05	–0.06	–0.06	−0.14**	–0.04	–		
16. Strength	0.36***	0.27***	0.24***	0.24***	0.26***	0.18***	0.20***	−0.25***	−0.27***	−0.13*	−0.14**	−0.15**	−0.21***	−0.12*	0.82***	–	
17. Optimism	0.32***	0.24***	0.22***	0.22***	0.24***	0.18***	0.18***	−0.18**	−0.24***	−0.14**	−0.13*	–0.10	−0.20***	−0.10*	0.60***	0.65***	–

T1 = Monday, T2 = Tuesday, T3 = Wednesday, T4 = Thursday, T5 = Friday, T6 = Saturday, T7 = Sunday, 1 = T1 positive affect, 2 = T1 positive affect, 3 = T1 positive affect, 4 = T1 positive affect, 5 = T1 positive affect, 6 = T1 positive affect, 7 = T1 positive affect, 8 = T1 negative affect, 9 = T1 negative affect, 10 = T1 negative affect, 11 = T1 negative affect, 12 = T1 negative affect, 13 = T1 negative affect, 14 = T1 negative affect, 15 = Tenacity, 16 = Strength, 17 = Optimism; *p < 0.05, **p < 0.01, ***p < 0.001.

### Trajectories of Daily Negative and Positive Affect

#### The Trajectory of Daily Negative Affect

A series of LGMMs were conducted to identify the trajectories of negative affect, with gender and SES as covariates. To identify the best-fitting model, five models with the number of profiles varying from one to five were conducted, whose fit indexes are presented in Table 3. As shown, the three-profile, four-profile, and five-profile models all contained a profile with only four (1.0%) participants. Therefore, the two-profile model was selected as the best-fitting model, as its entropy was second only to the highest value.

**TABLE 3:** Fit indexes of LGMMs for the negative affect.

**Model**	**K**	**Log(L)**	**AIC**	**BIC**	**aBIC**	**Entropy**	**LMR**	**BLRT**	**Probability**
1 Profile	16	−9159.654	18351.307	18414.806	18364.039				
2 Profiles	21	−9097.453	18236.905	18320.248	18253.616	0.893	>0.05	<0.0001	0.151/0.849
3 Profiles	26	−9043.643	18139.287	18242.473	18159.976	0.940	>0.05	<0.0001	0.803/0.010/0.187
4 Profiles	31	−9040.888	18143.776	18266.806	18168.444	0.682	>0.05	>0.05	0.010/0.197/0.284/0.509
5 Profiles	36	−9012.559	18097.118	18239.991	18125.765	0.744	>0.05	>0.05	0.010/0.189/0.417/0.325/0.059

**FIGURE 1:**
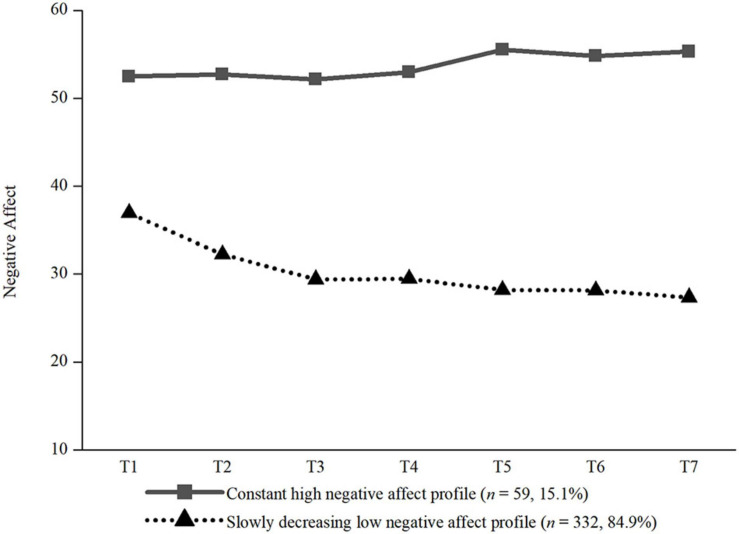
The two trajectories of negative affect. *N* = 391, T1 = Monday, T2 = Tuesday, T3 = Wednesday, T4 = Thursday, T5 = Friday, T6 = Saturday, T7 = Sunday.

The two profiles of negative affect can be seen in Figure 1. We labelled the two profiles based on their intercepts, slopes, and previous studies: (1) constant high negative affect profile, and (2) slowly decreasing low negative affect profile. The intercepts and slopes of every trajectory are presented in Table 4. The constant high negative affect profile (*n* = 59, 15.1%) had a higher initial level, and remained stable across the 7 days. The slowly decreasing low negative affect profile contained the majority of participants (*n* = 332, 84.9%), and decreased from a lower initial level across the 7 days.

**TABLE 4:** The intercepts and slopes of every group for the negative affect.

**Profile**	**Intercept**	**Slope**
Profile 1: constant high negative affect profile	51.074***	0.600
Profile 2: slowly decreasing low negative affect profile	32.427***	−0.988***

*p < 0.05, **p < 0.01, ** p < 0.001.

#### The Trajectory of Daily Positive Affect

A series of LGMMs were conducted to identify the trajectories of positive affect across the 7 days, with gender and SES as covariates. The fit indexes are presented in Table 5. As shown, the entropy of the three-profile model was the highest. Although the AIC and BIC were slightly higher than that of the four-profile model, the LMR-LRT and BLRT of the four-profile model were both not significant, indicating that the four-profile model was not better than the three-profile model. Therefore, the three-profile model was selected as the best-fitting model.

**TABLE 5:** Fit indexes of LGMMs for the positive affect.

**Model**	**K**	**Log(L)**	**AIC**	**BIC**	**aBIC**	**Entropy**	**LMR**	**BLRT**	**Probability**
1 Profile	16	−8286.896	16605.792	16669.291	16618.524				
2 Profiles	21	−8272.630	16587.259	16670.602	16603.970	0.789	<0.05	<0.0001	0.092/0.908
3 Profiles	26	−8252.595	16557.191	16660.377	16577.881	0.818	<0.05	<0.0001	0.087/0.046/0.867
4 Profiles	31	−8245.079	16552.158	16675.188	16576.827	0.789	>0.05	>0.05	0.785/0.056/0.113/0.046

**FIGURE 2:**
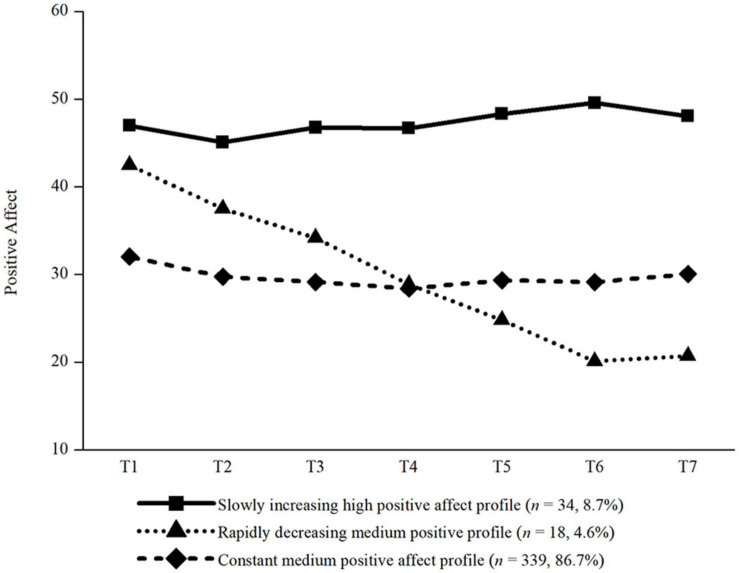
The three trajectories of positive affect. *N* = 391, T1 = Monday, T2 = Tuesday, T3 = Wednesday, T4 = Thursday, T5 = Friday, T6 = Saturday, T7 = Sunday.

The three profiles of positive affect can be seen in Figure 2. We labelled the three profiles based on their intercepts, slopes, and previous studies: (1) slowly increasing high positive affect profile, (2) rapidly decreasing medium positive affect profile, and (3) constant medium positive affect profile. The intercepts and slopes of every profile are presented in Table 6. The slowly increasing high positive affect profile (*n* = 34, 8.7%) had the highest initial level, and increased slightly across the 7 days. The rapidly decreasing medium positive affect profile (*n* = 18, 4.6%) had a slightly lower initial level, but decreased dramatically to the lowest level. The constant medium positive affect profile contained the majority of participants (*n* = 339, 86.7%), whose levels of positive affect remained medium across the 7 days.

**TABLE 6:** The intercepts and slopes of every group for the positive affect.

**Profile**	**Intercept**	**Slope**
Profile 1: slowly increasing high positive affect profile	42.875***	0.383*
Profile 2: rapidly decreasing medium positive affect profile	40.525***	−3.546***
Profile 3: constant medium positive affect profile	29.898***	−0.157

*p < 0.05, ***p < 0.001.

### Resilience and Gender as Predictors of Daily Emotion Trajectories

#### Daily Negative Affect

Using constant high profile as the reference category, logistic regressions were conducted to examine whether and to what extent three dimensions of resilience and gender affected the probability of trajectory membership in daily negative affect.

As shown in Table 7, the optimism dimension of resilience played a significant role in distinguishing different trajectory memberships of negative affect. Compared with constant high profile, college students with high levels of optimism had a higher probability of having a slowly decreasing low profile [OR = 1.08, 95% CI = (1.01, 1.15)]. The predictive effects of tenacity, strength, gender, and the interactions between the three dimensions of resilience and gender on negative affect trajectory membership were not significant (*p*s >0.05).

**TABLE 7:** Multinomial logistic regression predicting negative affect trajectory class membership (Odds ratios).

	**Profile 2 vs. Profile 1**	
	OR	95%CI
Tenacity	−	−
Optimism	1.08*	[1.01, 1.15]
Strength	−	−
Sex (male = 1, female = 0)	−	−
Tenacity * Sex	−	−
Optimism * Sex	−	−
Strength * Sex	−	−

*Table 7 presents odds ratios (OR) for binary logistic regression. Profile 1 = constant high negative affect profile; Profile 2 = slowly decreasing low negative affect profile. Profile 1 and males are the reference categories. The non-significant variable and interactions that were dropped from the final analysis are indicated by ns;* *p < 0.05.

#### Daily Positive Affect

Using slowly increasing high and constant medium positive affect profiles as the reference categories, logistic regressions were conducted to examine whether and to what extent the three dimensions of resilience and gender influenced the probability of trajectory membership in daily positive affect.

As shown in Table 8, the optimism dimension of resilience and gender played a significant role in distinguishing different trajectory memberships of positive affect. Compared with a constant medium profile, college students with high levels of optimism had a higher probability of having a slowly increasing high profile [OR = 1.45, 95% CI = (1.22, 1.72)] and rapidly decreasing medium profile [OR = 1.37, 95% CI = (1.10, 1.70)]. Compared with a constant medium profile, males were more likely than females to have a slowly increasing high profile [OR = 0.33, 95%CI = (0.16, 0.72)]. Compared with a slowly increasing high profile, females were more likely than males to have a rapidly decreasing medium profile [OR = 3.56, 95% CI = (1.06, 11.98)]. However, the predictive effects of tenacity, strength, and the interactions between the three dimensions of resilience and gender on positive affect trajectory membership were not significant (*p*s >0.05).

**TABLE 8:** Multinomial logistic regression predicting positive affect trajectory class membership (Odds ratios).

	**Profile 1 vs.**		**Profile 2 vs.**		**Profile 2 vs.**	
	**Profile 3**		**Profile 3**		**Profile 1**	
	**OR**	**95%CI**	**OR**	**95%CI**	**OR**	**95%CI**
Tenacity	−	−	−	−	−	−
Optimism	1.45***	[1.22, 1.72]	1.37**	[1.10, 1.70]	−	−
Strength	−	−	−	−	−	−
Sex (male = 1, female = 0)	0.33**	[0.16, 0.72]	−	−	3.56*	[1.06, 11.98]
Tenacity * Sex	−	−	−	−	−	−
Optimism * Sex	−	−	−	−	−	−
Strength * Sex	−	−	−	−	−	−

*Table presents odds ratios (OR) for multinomial logistic regression. Profile 1 = slowly increasing high positive affect profile; Profile 2 = rapidly decreasing medium positive affect profile; Profile 3 = constant medium positive affect profile. Profile 3, Profile 1 and males are the reference categories. The non-significant variable and interactions that were dropped from the final analysis are indicated by ns;* *p < 0.05, **p < 0.01, ***p < 0.001.

## Discussion

In the past year, a growing body of studies have focused on college students’ daily emotional experiences caused by the COVID-19 pandemic (Alyami et al., 2020; Brooks et al., 2020; Wang C. et al., 2020). However, these studies mainly paid attention to the intensity and frequency of the pathological outcomes of college students during COVID-19. Little attention was paid to their daily emotion, including daily negative and positive affect. Moreover, far less was known about how their daily negative and positive affect varied from time to time and from people to people. Therefore, the current study shed light on the heterogeneous trajectories of college students’ daily negative and positive affect during the COVID-19 pandemic, and the prediction power of resilience on the trajectories.

The LGMMs conducted in this study yielded two profiles of daily negative affect: (1) constant high negative affect profile, and (2) slowly decreasing low negative affect profile. Among them, 59 (15.1%) college students were assigned to the constant high negative affect profile, while 332 (84.9%) college students were assigned to the slowly decreasing low negative affect profile. In other words, although a small minority of college students showed constant high negative affect, most students’ initial levels of negative affect were low, and showed a decreasing trend over the 7 days. Previous studies related to COVID-19 and other similar epidemics showed that various negative emotions including depression, anxiety, and panic appeared and remained constant during the outbreak of an epidemic (Maunder et al., 2003; Xiang et al., 2020). However, different from these studies, the findings of the present study indicated that the emotional consequences of COVID-19 were of great diversity, and did not have to be continuously negative, which was consistent with results from Tausczik et al. (2012), who found that anxiety decreased significantly over 2 weeks during the H1N1 outbreak. This seemed to suggest a new understanding of college students’ emotional consequences of COVID-19, which was of great value in extending the existing literature.

For positive affect, three profiles were found: (1) slowly increasing high positive affect profile, (2) rapidly decreasing medium positive affect profile, and (3) constant medium positive affect profile. Among them, 34 (8.7%) college students were assigned to the slowly increasing high positive affect profile. Eighteen (4.6%) students were assigned to the rapidly decreasing medium positive affect profile. And 339 (86.7%) were assigned to the constant medium positive affect profile. This indicated that negative affect and positive affect were two different dimensions. During the COVID-19 pandemic, college students experienced not only negative affect, but also positive affect in their daily life, the change patterns of which varied from person to person. These findings were partially consistent with the results from Brondino et al. (2020), who examined the different change patterns of positive affect across 7 days in normal life before COVID-19, and also found three trajectories: a trajectory with constant high levels of positive affect, a trajectory with an increasing growth of positive affect across the 7 days, and a trajectory with a decreasing growth. According to the results from Brondino et al. (2020), most participants reported constant high levels of positive affect. However, different from them, the findings of the current study indicated that most students’ levels of positive affect remained medium over the 7 days, which might be due to the emotional consequences of the COVID-19 pandemic. These findings might make a great contribution to the creation of prevention and intervention measures in promoting college students’ psychological health development under the threat of pandemic diseases. For instance, researchers and practitioners should pay more attention to identifying students’ profiles of both negative and positive affect trajectories, and adopt targeted measures accordingly.

Predictions concerning the three different dimensions of resilience in college students’ daily emotional experience were supported in the present study. According to the findings of the present study, college students with higher levels of optimism were more likely than those with lower levels of optimism to belong to the slowly increasing high and rapidly decreasing medium positive affect profiles and slowly decreasing low negative affect profile. But the prediction of tenacity and strength were not significant. Consistent with previous studies that focused on the role of resilience in emotion (Hou J. et al., 2020; Kang et al., 2020; Ran et al., 2020; Wang J. et al., 2020), this study provided further evidence that optimism was an important resource and could mitigate the negative impact of public health emergencies and stress on individual emotion. A possible explanation was that the optimism dimension of resilience emphasized a positive attitude in adverse situations (Hou J. et al., 2020). So college students with high levels of optimism were inclined to use valid coping strategies to regulate emotion under negative situations, such as home isolation, online learning and academic pressure (Wang Y. et al., 2016; Alessandri et al., 2020). However, tenacity offered a strong sense of power and perseverance in the face of setbacks. It described a person who had a sense of control when facing difficulties. Meanwhile, strength mainly reflected a person with the ability to recover from adverse experiences. All in all, the two dimensions of resilience mainly represented a person that could flexibly cope with adversity and had coping methods to face challenges (Yu and Zhang, 2007; Wang A. et al., 2016; Hou T. et al., 2020), and were less involved in individual emotional experiences. Thus, optimism should be taken into consideration when planning prevention and intervention efforts to promote individual positive emotional development.

Besides, the current study found that females were more likely than males to belong to the rapidly decreasing medium positive affect profile, while males were more likely than females to belong to the slowly increasing high positive affect profile. This is consistent with previous studies (Neumann et al., 2011; Gur et al., 2012; Marceau et al., 2012; Hu et al., 2014), indicating that females were more prone to experience emotional problems. One possible explanation may be attributed to the fact that COVID-19, as a potential stressor, increased vulnerability for females, while males could timely adjust their internal emotion to face the issue with a positive attitude (Boals, 2010; Mauvais-Jarvis et al., 2020). Another possible explanation may be that females paid more attention to their emotions (Neumann et al., 2011), and they were more likely to respond to stress by turning their feelings inward (Steinberg, 2014). This leads to a higher decrease in females’ daily positive affect than males. Overall, as suggested by these findings, more attention should be paid to females’ physical and mental health during the COVID-19 pandemic.

This study has some limitations that point to directions for future research. Firstly, we did not collect resilience for longitudinal data to explore its changes over time. That is to say, we could not draw a causal conclusion about the relation between resilience and daily emotional experiences. Secondly, the distribution of the participants was asymmetrical in the provinces of China. Thus, we must be cautious to generalize the results to other provinces which had different COVID-19 incidence rates. Finally, the influence of other negative life events on participants’ emotion was not controlled. So it would be necessary to exclude the impact of other negative life events in future research.

Despite these limitations, this study provides a relevant contribution to the existing research and has important implications for practice. First, to our knowledge, this is the first study to separately investigate the heterogeneity growth trajectories of daily negative and positive affect during the COVID-19 outbreak in college students. Second, this study used a person-centred approach to focus on individuals’ heterogeneity of trajectories in daily emotion. It distinguished the subgroups of college students who were more sensitive to the COVID-19 outbreak and more resilient to the stressful event. This provides valuable and practical information for the development of intervention programs for vulnerable college students. In addition, these findings suggested that facilitating resilience, especially optimism, may be effective for promoting college student’s positive affect and reducing negative affect. These findings provide a major goal for intervening in individuals’ emotional fluctuation during COVID-19.

## Data Availability Statement

The raw data supporting the conclusions of this article will be made available by the authors, without undue reservation.

## Ethics Statement

The studies involving human participants were reviewed and approved by the Institutional Review Board of Shandong Normal University. The patients/participants provided their written informed consent to participate in this study.

## Author Contributions

LZ, LW, and YL have contributed equally to this work and share first authorship. All authors listed have made a substantial, direct and intellectual contribution to the work, and approved it for publication.

## Conflict of Interest

The authors declare that the research was conducted in the absence of any commercial or financial relationships that could be construed as a potential conflict of interest.
